# Intracranial Hypotension Syndrome: The Importance of Neurointensive Care

**DOI:** 10.7759/cureus.42673

**Published:** 2023-07-30

**Authors:** Carolina Roriz, Maria Ana Canelas, Eduarda Pereira

**Affiliations:** 1 Intensive Care Unit, Centro Hospitalar de Leiria, Leiria, PRT; 2 Intensive Care Unit, Centro Hospitalar de Vila Nova de Gaia/Espinho, Gaia, PRT; 3 Neurocritical Care Unit and Intensive Care, Hospital São João, Porto, PRT

**Keywords:** multimodal neuromonitoring, intracranial hypotension, cerebrospinal fluid leak, critical care, laminectomy

## Abstract

Surgical procedures involving the spine can result in various complications, including vascular, nerve root and dura mater injury, surgical wound infection, and hematoma formation. Unintentional durotomy is a frequent complication of these procedures (up to 17%). Two clinical cases are reported in which the occurrence of epileptiform activity in the form of generalized tonic-clonic seizures after instrumentation of the dorsal and lumbar spine raised suspicion of cerebrospinal fluid (CSF) fistula. In both cases, the diagnostic suspicion and early approach allowed for the adoption of a timely medical and surgical plan, with the aim of reducing the volume of lost CSF as well as the potential neurological dysfunction resulting from this surgical complication.

## Introduction

Unintentional durotomy is a frequent complication of spinal surgery (17%) [1], which can lead to postoperative intracranial hypotension-associated venous congestion and pseudohypoxic brain swelling in a small proportion of patients [2]. Wound drainage systems and a rapid loss of cerebrospinal fluid (CSF) are usually present [2]. This case report highlights the clinical cases of two female patients, aged 76 and 83 years, who underwent complex spinal surgeries. Complications arose postoperatively, including generalized tonic-clonic seizures and cerebral edema. CSF hypotension was suspected as the underlying cause. Detailed radiological evaluations and neuroprotective measures were undertaken to manage the complications effectively. Both patients underwent successful surgical exploration to address the issue and experienced a good recovery without focal neurological deficits. This report emphasizes the importance of early recognition and management of complications in complex spinal surgeries for improved patient outcomes.

## Case presentation

**Figure 1:**
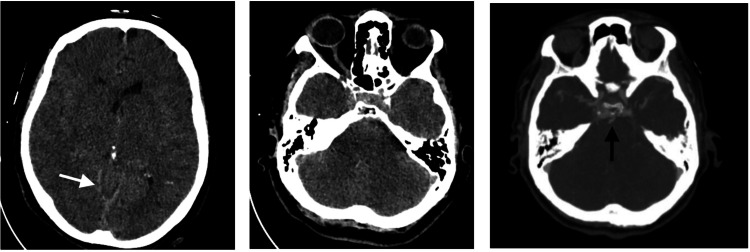
Brain computed tomography of patient 1 There is a diffuse loss of differentiation between gray matter and white matter, both supratentorial and infratentorial, with reduced dimensions of the ventricular system and significant protrusion of the cerebellar tonsils into the region of the foramen magnum. There is also a reduction in amplitude and diffuse and symmetric accentuation of hyperdensity in the cisternal spaces as well as the cerebral falx and cerebellar tentorium, with the appearance of "pseudo-subarachnoid hemorrhage" (white arrow). All these aspects suggest the presence of diffuse cerebral edema. Angiography study shows engorgement of venous structures (black arrow), with prominence of the silhouettes of the cavernous sinuses and venous plexus in the craniocervical and upper cervical transition.

We present the clinical cases of two female patients aged 76 and 83 years (patient 1 and patient 2, respectively) who underwent posterior surgical decompression of the D9 vertebra, curettage of the same vertebral body, and bilateral L5 laminectomy and foraminectomy with autologous graft placement, respectively. Both patients had a history of medicated arterial hypertension. The surgical procedures were performed in a prone position under balanced general anesthesia. The general anesthesia was induced with propofol, fentanyl, and rocuronium, while sevoflurane was used for anesthetic maintenance. Regarding patient 1, the surgical intervention proceeded without complications, particularly from a respiratory and hemodynamic standpoint. At the anesthetic emergence, the patient developed generalized tonic-clonic seizures, which were reverted after intravenous administration of a benzodiazepine. Anesthesia was induced again for further evaluation. As for patient 2, at the end of the surgical intervention, a decrease in the capnography value was observed along with hypotension, which was reverted after the administration of 30 mg of intravenous ephedrine. At the anesthetic emergence, she presented spontaneous eye opening with conjugated gaze deviation to the right and motor response in flexion followed by generalized tonic-clonic seizures, which were reverted after intravenous benzodiazepine administration. The intraoperative blood losses were negligible, and no hydroelectrolytic disturbances were observed. Both patients underwent a brain computed tomography (CT) scan, which revealed diffuse loss of differentiation between gray and white matter and projection of cerebellar tonsils in the region of the foramen magnum, suggestive of diffuse cerebral edema. In the case of patient 1, the angiography study also showed engorgement of venous structures, with prominence of the silhouettes of the cavernous sinuses and venous plexus in the craniocervical and upper cervical transition as well as a globular appearance of the pituitary gland (Figure 1).

In the case of patient 2, there was also evidence of bilateral intraparenchymal hemorrhagic foci in the left anterior and right occipital regions as well as in the left ambient and interpeduncular cisterns in the right fronto-basal region. Based on these data, the diagnostic hypothesis of CSF hypotension secondary to unintentional dural tears was raised. Both patients were admitted to the neurocritical care unit for neuroprotective measures, including monitoring of the depth of sedation with bispectral index, cerebral saturation with near-infrared spectroscopy, and etiological investigation to improve neurological outcomes. In the case of patient 1, an intracranial pressure catheter was placed to monitor optimal cerebral perfusion pressure through the cerebral reactivity index (PRx). During the ICU stay, a liquid with a similar appearance to CSF was observed on the surgical drain in patient 2, which was not on suction, but the imaging documentation of CSF fistula was not possible due to the artifacts from the metallic material. On the other hand, in patient 1, the lumbar spinal MRI allowed the identification of an extracanal collection with similar intensity to CSF at the surgical site, with craniocaudal extension from posterior levels of the L3-L4 intervertebral disc to posterior levels of the upper third of the S1 body and posterior planar extension involving the L4 spinous process and reaching cutaneous planes at the L4 level.

**Figure 2:**
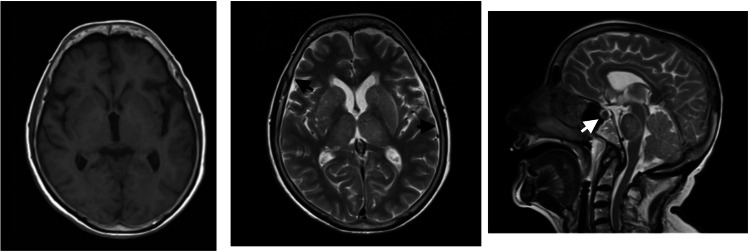
Brain magnetic resonance imaging of patient 2 Extensive edematous changes with a marked predominance of vasogenic edema at the level of the basal ganglia, radiated crowns, and frontal and occipital white matter bilaterally in the pontine region and the cerebellar hemispheres, with the presence of associated cytotoxic edema areas, are observed. Changes consistent with a pseudohypoxic pattern are described in the context of spinal surgical intervention. There was also an engorgement of the pituitary gland (white arrow), diffuse thickening, and pachymeningeal enhancement (black arrows); these findings are compatible with a context of cerebrospinal fluid hypotension.

In both cases, prior to the suspension of sedoanalgesia, a brain MRI was performed for the evaluation and stratification of the lesions described upon admission. Therefore, the cerebral MRI was performed four days and two days after surgical intervention in patients 1 and 2, respectively. In patient 2, edematous changes of mixed nature were observed, with the predominance of vasogenic edema in the basal nuclei, radiating crowns, bilateral frontal and occipital white matter as well as cytotoxic edema in the lentiform nuclei, thalamus, and cerebellar vermis. There was also swelling of the pituitary gland, diffuse thickening, and pachymeningeal enhancement (Figure 2).

On the other hand, in patient 1, no additional CSF loss was observed through the surgical wound; the imaging findings demonstrated the absence of diffusion restriction areas in the thalamus and a reduction of areas of subtle hyperintensity in long TR sequences in the internal and external capsules, bilateral frontal and parietal white matter, dentate nuclei and posterior aspect of the pons, and bilateral midbrain tegmentum, and the cerebral tonsils were already in normal position.

In both patients, a new surgical exploration was performed by the orthopedic team to identify and close the dural tear. In the postoperative period, progressive head bed elevation up to 45º was performed, and no suspected drainage by the wound drains was observed, making it possible to safely suspend sedation and proceed with extubation without complications. The patients were discharged to the orthopedic ward without focal neurological deficits.

## Discussion

Postoperative intracranial hypotension is a rare complication after spinal surgery [3]. It usually occurs after the loss of significant amounts of CSF following spine or cerebral surgery with intended or unintended opening of the dura mater. Unexpected postoperative deterioration and failure to regain consciousness, sometimes accompanied by seizure activity, are typical and may result in death or leave the patient impaired permanently. Multiple etiologies of CSF leak due to dural tears during surgeries were reported, including direct trauma, excessive nerve root traction, and misplaced instrumentation [4]. In our cases, the etiology of CSF leak was direct trauma. The risk factors that can precipitate this complication include older age, female sex, previous surgery with scar formation, corrective vertebral osteotomy, degenerative spondylolisthesis, and radiotherapy that can impair tissue healing [4]. Therefore, surgical decision-making must require a systematic preoperative clinical evaluation and should be performed in order to stratify risks and guide decision-making for obtaining the best possible clinical results at lower risk [5].

According to the Monro-Kellie doctrine, the decrease in CSF volume is compensated by the increase in the volume of low-resistance structures, namely cerebral and peri-pituitary veins and dural sinuses [6]. As the cerebral volume is probably invariable, compensation occurs through an increase in cerebral blood volume [6]. Imaging signs of CSF are represented by diffuse non-nodular thickening of the pachymeningeal layer, subdural collections (often bilateral), craniocaudal displacement of the brain (described in the Anglo-Saxon literature as sagging), and venous and pituitary engorgement [2].

Al-Gethami et al. described a man who underwent cervical decompression, with an accidentally identified and corrected durotomy during the intraoperative period. In this patient’s case, seizures occurred during the recovery. A head CT scan revealed pneumoencephalus in the subarachnoid space, right subdural hematoma, and subarachnoid hemorrhage. The patient made a complete recovery without subsequent epileptiform activity [4] similar to the patients described in our report. Parpaley et al. also described two patients presenting with a clinical and radiological picture of pseudohypoxic brain swelling after spinal surgery. In the first patient, bilateral basal ganglia damage occurred after thoracic spondylodiscitis surgery, manifested by epileptic seizures and coma lasting one week postoperatively with subsequent recovery. The second patient suffered basal ganglia and cerebellar and brainstem infarction after lumbar spondylodiscitis surgery, resulting in death. In both patients, intraoperative fluid leakage was observed, and epidural suction drainage with CSF loss occurred [7]. Sporns et al. conducted systematic PubMed-based research of the literature to study the variety and frequency of the reported symptoms of post-traumatic postoperative loss of CSF. All 15 cases (of a total of 27 reported) in which a negative pressure suction device had been applied showed severe neurological and radiological symptoms such as coma or brain herniation and intracranial hemorrhage. In all cases, patients recovered rapidly after the removal of the suction device [8].

The classic imaging finding of intracranial hypotension is the presence of smooth pachymeningeal enhancement. Other brain MRI findings include downward displacement of the cerebellum resulting in effacement of the prepontine and prechiasmatic cisterns, flattening or tenting of the optic chiasm, subdural effusions, enlargement of the pituitary, and engorgement of the dural sinuses. MRI findings of the spine include epidural fluid collections, dilation of the epidural venous plexus, and prominent spinal cord veins [9]. In the reported cases, some of those findings were presented. Although the exact mechanism of intracranial hemorrhage is not known, it is postulated that intracranial hematomas development is related to a persistent decrease in intracranial pressure due to loss of CSF volume leading to stretching and then tearing of the bridging veins [4].

Finally, admission to the intensive care unit with the possibility of neurointensive care allowed for an adequate approach to these patients as there is evidence that admission to an intensive care unit specialized in brain and spine injury is associated with improved outcomes [10]. This allowed the patient to receive neuroprotective measures and at the same time enabled surgical correction of the dural tear in a brief manner, with excellent neurological outcomes.

## Conclusions

In conclusion, this case report presented two clinical cases highlighting the occurrence of intracranial hypotension syndrome as a complication following iatrogenic durotomy. The occurrence of generalized tonic-clonic seizures, cerebral edema, and CSF leak emphasized the need for vigilance and prompt intervention. Radiological evaluations played a crucial role in identifying the imaging hallmarks of intracranial hypotension, allowing for timely targeted management strategies. Neuroprotective measures and surgical exploration to repair the durotomy prevented further neurological dysfunction. Furthermore, the multidisciplinary approach and intensive care unit admission played significant roles in providing optimal care.
